# The history of risk: a review

**DOI:** 10.1186/s13017-017-0125-6

**Published:** 2017-03-14

**Authors:** Philip F. Stahel, Ivor S. Douglas, Todd F. VanderHeiden, Sebastian Weckbach

**Affiliations:** 1 Department of Orthopaedics, University of Colorado, School of Medicine and Denver Health Medical Center, 777 Bannock Street, Denver, CO 80204 USA; 2 Department of Neurosurgery, University of Colorado, School of Medicine and Denver Health Medical Center, Denver, CO 80204 USA; 3 Department of Medicine, Division of Pulmonary Sciences and Critical Care, University of Colorado, School of Medicine and Denver Health Medical Center, Denver, CO 80204 USA; 4 Department of Orthopaedics, University Hospital Ulm, D-89081 Ulm, Germany

**Keywords:** Risk, History, Probability theory, Risk management, Decision-making

## Abstract

In the USA alone, around 22 million patients annually discuss the need for surgical procedure with their surgeon. On a global scale, more than 200 million patients are exposed to the risk of undergoing a surgical procedure every year. A crucial part of the informed consent process for surgery is the understanding of risk, the probability of complications, and the predicted occurrence of adverse events. Ironically, risk quantification, risk stratification, and risk management are not necessarily part of a surgeon’s core skillset, considering the lengthy surgical training curriculum towards technical excellence. The present review was designed to provide a concise historic perspective on the evolution of our current understanding of risk and probability, which represent the key underlying pillars of the shared decision-making process between surgeons and patients when discussing surgical treatment options.

## Background

It is a fascinating and uncommonly appreciated fact that our current “modern times,” characterized by the technological advances that shape and define our daily lives, originate from the introduction of a Hindu-Arabic numbering system in Italy in the early 1200s. The ability of society to utilize numerals for arithmetic calculus propelled our understanding of odds, risk, and probability, starting from the time of the Renaissance in Europe to our current modern age in a global perspective [1]. Most readers are likely not aware that almost all significant inventions, innovations, and developments in science, economics, technology, and health care in the past 200–300 years originated from the ability to predict future events and to make conscientious, balanced decisions on the risk and probability of our actions [2]. The revolutionary “risk movement” was capitalized during the Renaissance and brought to fruition in the sixteenth and seventeenth century in France, Italy, and Germany by a few selected risk-takers who dared to think outside of the religious boundaries of their time [2]. These heuristic thinkers and pioneers showed courage in defying the state-of-the-art rules which had historically been defined and enforced by society and religion [3]. Pragmatically speaking, what distinguishes ten-thousands of years of the history of humankind from our current “modern times” has been almost exclusively driven by the introduction of probability theory and risk management [2]. Before the sixteenth century, humankind was guided by their faith and belief in fate and divine intervention. The incoming understanding and new mastery of risk, which is in essence owed to the introduction of the Hindu-Arabic numbering system to Italy in the early 1200s, led to the evolution of our modern society in the twenty-first century [2]. Impressively, the precursor of our modern numerals dates back to the brilliant Indian mathematician Brahmagupta, who also introduced the number “zero” in his encompassing treatise “The Opening of the Universe” (*Brāhmasphutasiddhānta*) in 628 AD. The age of the crusades in the Middle East allowed for Western civilization to collide with the Arabic empire during the Medieval period [4]. The Arabs had previously introduced the Hindu numbering system after their invasion of India [4]. Dice games that were brought to Europe through the crusaders set the basis for our modern game of *Craps* [5]. Interestingly, *Al-Zahr* (the Arabic word for dice) provided the root for our modern designation of “hazard.” As surgeons expose their patients to hazard/risk on a daily basis, it appears pertinent to reflect on the historic origin of probability theory and risk management, to improve the understanding of these crucial entities as part of the surgical decision-making.

## Fibonacci’s arithmetic revolution

**Fig. 1:**
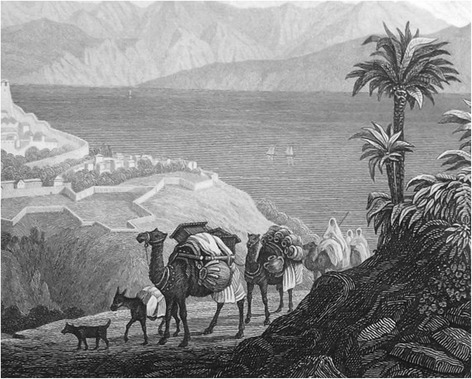
The historic Algerian port city of Bugia (*Béjaïa*), where Fibonacci learned the Hindu-Arabic numerals from Arabic mathematicians and merchants in the late twelfth century. Source: “Atlas Mountains and City of Bugia, Algeria,” 1870 (public domain)

**Fig. 2:**
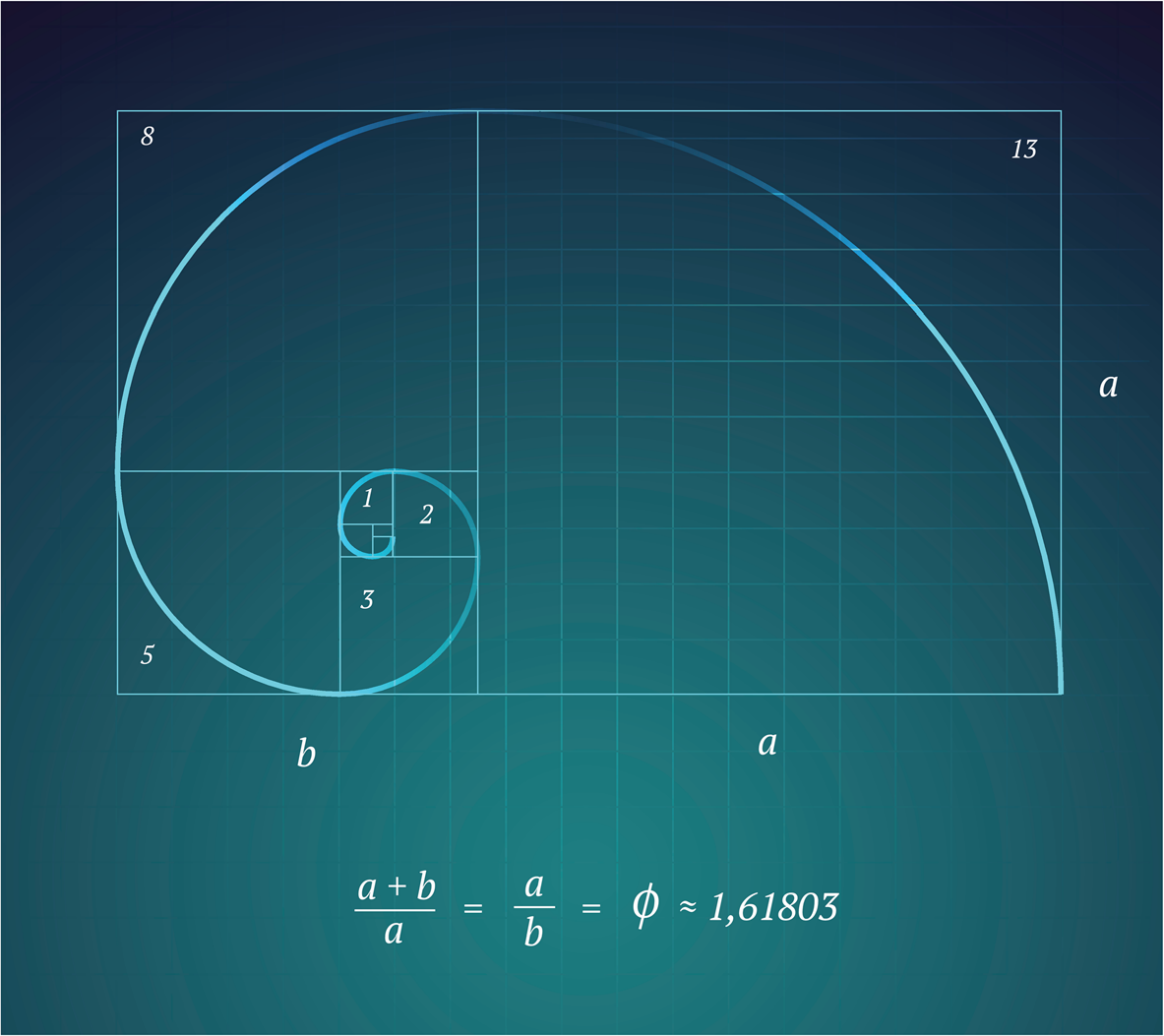
The Fibonacci sequence as the underlying solution of the “golden mean.” Reprinted with permission (iStock/Getty Images, ID 471739880, © by mastaka 2015)

The history of numbers in Western society was initiated by the impressive work of a young Italian, Leonardo Pisano, who published the “Book of Calculation” (*Liber abbaci*, frequently misquoted as *Liber Abaci*) in 1202 [6]. Pisano’s legacy is commonly known under his nickname Fibonacci, which is derived from the Latin *filius Bonacci* and extrapolates to “The son of the Bonacci family” [7]. Fibonacci learned the Hindu-Arabic numbering system from Arabic mathematicians and merchants during his trip to Northern Africa when he visited his father in the port city of *Bugia* (now *Béjaïa*, Algeria) as a teenager (Fig. 1). The new insights led the young Italian to question the value of the traditional Roman numbering system that had been exclusively based on plain letters and therefore did not allow for mathematical calculations. Intriguingly, the traditional Roman numeral system was devoid of a symbol for “zero,” as the number zero was not practically relevant in ancient life [8]. The major shortcoming of the lack of the numeral zero relates to the impediment of performing calculations in multiples of tens, hundreds, and etc., and the inability to calculate with negative numbers, both of which represent prerequisites for modern mathematics, technology, and science [8]. Fibonacci’s historic legacy is represented by his ability to define the “golden mean” (or “golden ratio”), a previously unresolved enigma that dated back to the times of the ancient Greek philosophers and mathematicians (Fig. 2). At Fibonacci’s times, understanding the golden mean was considered as close as unifying the principles of mathematics and science with nature and God [9].

The Fibonacci sequence is one of the groundbreaking new insights in his *Liber abacci* [6]. Most readers may recognize Fibonacci’s name from Dan Brown’s best-selling novel, *The Da Vinci Code*, where a dying man in the opening scene scrawled Fibonacci’s sequence in invisible ink on the floor of the Louvre museum in Paris [10]. The intriguing origin of the Fibonacci sequence, however, is scarcely known: As a young man, Fibonacci was challenged with the ancient task of calculating how many rabbits would be born within 1 year, originating from a single pair of rabbits. His calculation was based on the assumption that each rabbit pair will produce another pair of rabbits every month, and rabbit pairs start breeding when they are 2 months old. Fibonacci described the problem in chapter 12 of his *Liber abbaci*, as such [6]: “A certain man had one pair of rabbits together in a certain enclosed place, and one wishes to know how many are created from the pair in one year when it is the nature of them in a single month to bear another pair, and in the second month those born to bear also.”

**Fig. 3:**
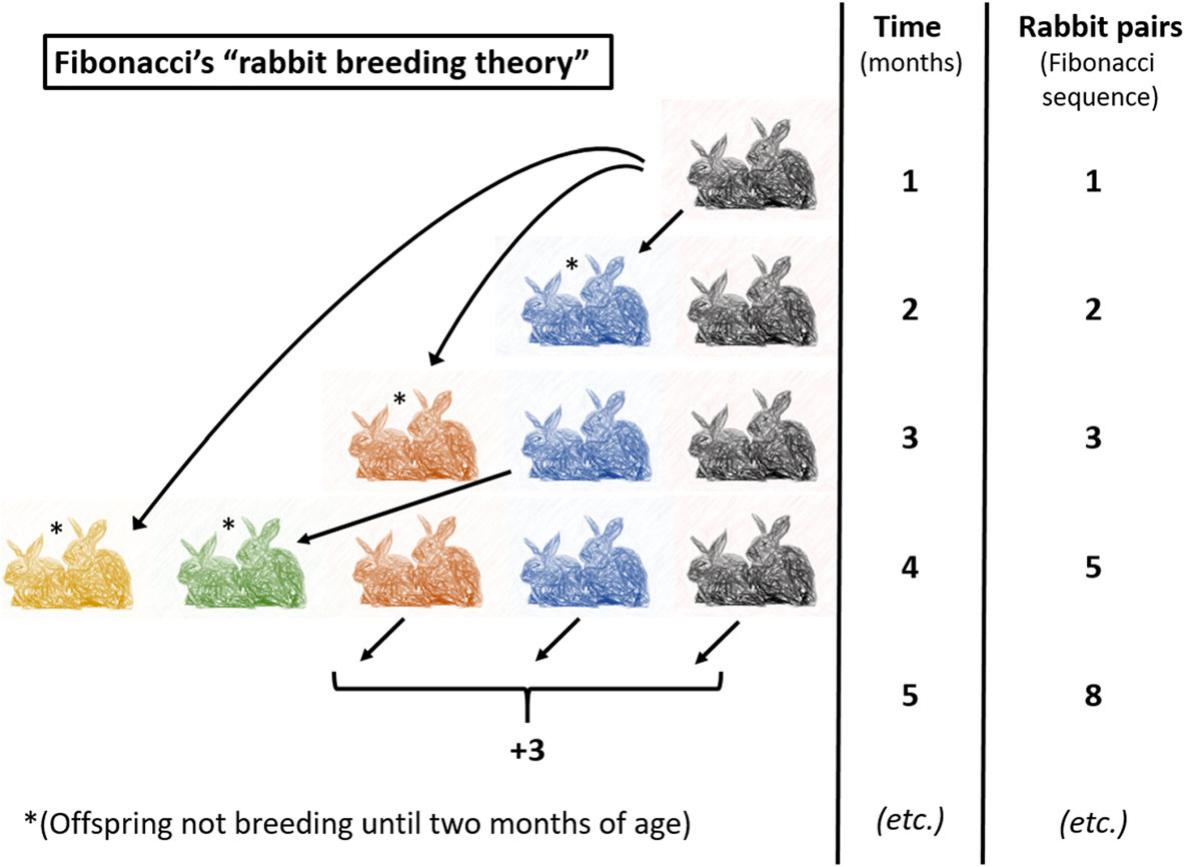
Schematic explanation of the “rabbit breeding theory” as the origin of the Fibonacci sequence

Fibonacci’s solution to the “rabbit breeding theory” [7] set the foundation of a historic mathematical miracle (Fig. 3). In brief, there is one pair of rabbits in 1 month, one additional pair of rabbits as first offspring from the original pair, three in 3 months (an additional couple from the original pair), five in 4 months (with the first offspring now breeding as well), followed by eight, 13, 21, 34, 55, 89, 144, and a total of 233 pairs of rabbits at the end of the first year. This sequence of numbers represents the essence of the Fibonacci sequence, where each number represents the sum of the two preceding numbers. The mathematical magic about this simple series of numbers is that the “divine proportion” (golden ratio) sought by Aristotle in ancient Greece as a philosophical concept of a “desirable middle between two extremes, one of excess and the other of deficiency” is calculated by dividing any number in the Fibonacci sequence, after number 144, by its preceding number. The result will always be 1.618—the mathematical constant PHI (Fig. 2) [11]. In general terms, the golden mean implies a perfect moderate position that avoids extremes, e.g., as a ratio between the two divisions of a line such that the ratio of the smaller to the larger is identical to the ratio of the larger to the sum of both lines. We encounter the golden ratio every day in arts, culture, religion (for example in the cross of Christ), and in multiplicity of phenomena in mathematics, science, and nature, including in plants and animals [11, 12].

## How gambling inspired modern probability theory

Fibonacci’s seminal work from the early 1200s was met by intense resistance for several hundred years. It was only in the early Renaissance in Italy during the late 1400s and early 1500s that mathematicians reflected back on Fibonacci’s theories and the Hindu-Arabic numbering system. Two renowned gamblers, the Franciscan monk Luca Pacioli and the Italian physician Girolamo Cardano from Milan, provided a formal mathematical analysis of the probabilities in dice throwing. Both pioneers authored groundbreaking books in the field [13, 14]. Pacioli, who was a good friend of Leonardo da Vinci, attempted to answer the historically unresolved question on how to divide the stakes in an incomplete game, when one of the two gamblers is leading but the game has not yet been won. Pacioli’s work was the first formal quantification of risk. Luca Pacioli is also known as the earliest known writer on double-entry bookkeeping [15]. Girolamo Cardano’s work *Liber de ludo aleae* in 1564 (“The Book on the Game of Dice”—published posthumously in 1663) was the first serious effort to elaborate on statistical principles of probability [14]. Cardano’s legendary quote from the book is the pragmatic conclusion that “the greatest advantage from gambling comes from not playing it at all.” His innovative work was the first publication on risk management and analysis of the laws of probability [14]. Subsequent to the publications by Pacioli and Cardano, it took another century until the great mathematicians and philosophers Blaise Pascal in France and Gottfried Wilhelm Leibniz in Germany introduced the first systematic mathematical method for calculating probability of future events. Pascal and Leibniz provided different mathematical solutions to Pacioli’s classic problem of how two players split the stakes in a game when they leave a game uncompleted [16, 17].

**Fig. 4:**
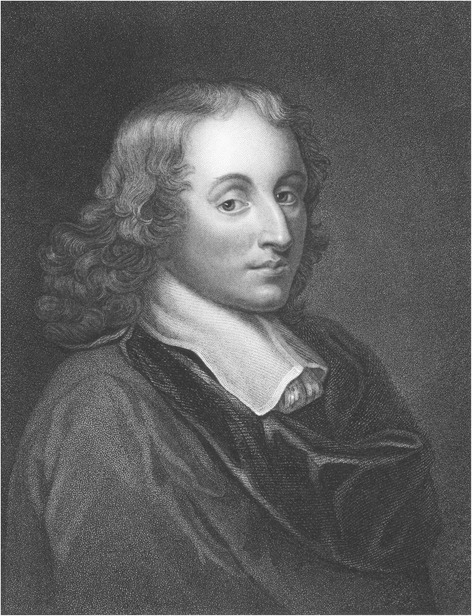
Blaise Pascal (1623–1662). Reprinted with permission (iStock/Getty Images, ID 97011251, © by GeorgiosArt 2010)

**Fig. 5:**
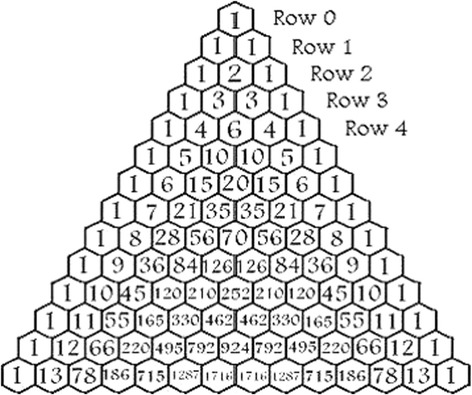
Pascal’s triangle for probability calculation. Reprinted with permission (licensed under the Creative Commons Attribution 4.0 International license)

Blaise Pascal, the “father of the modern theory of decision-making” (Fig. 4), constructed a systematic method for analyzing the probability of future outcomes using a simple triangle [18]. Pascal’s triangle was published in his seminal work *Traité du triangle arithmétique* in 1653 and introduced a pragmatic and simple solution to the historic question on how to divide the stakes in an unfinished game [18]. In the triangle, each number is the sum of the two numbers directly above (Fig. 5). The first and last number in each row are always a number 1, since the non-depicted, omitted numbers next to each number 1 represent a zero (0 + 1 = 1). The basic concept of Pascal’s triangle is that the top number in row 0 shows a probability of an event that cannot fail to happen, with only one possible outcome (100%). This is ensued by a 50:50 probability in row 1 (1-1), a 25:50:25 probability in row 2 (1-2-1), and etc.

An illustrative example based on coin tosses in the classic “heads or tails” bet may help more easily understand the utility of Pascal’s triangle for probability calculation:

- ✓ What is the probability of rolling two heads with two coin tosses? Row 2 is representative of 2 coin tosses with 4 (2^2^) possible outcomes (1-2-1). There is only one option for throwing two heads, with a probability of 1/4 or 25% (Table 1).
- ✓ What is the probability of tossing at least two heads with four coin tosses? Row 4 is representative of four coin tosses with 16 (2^4^) possible outcomes (1-4-6-4-1). There are 1 + 4 + 6 options for tossing at least two heads, i.e., 11/16 or 68.75%. Based on the same example in row 4, the probability of throwing four heads with four coin tosses is 1/16 or 6.25% (Table 1).

**Table 1:** Examples of probability calculations for coin tosses, based on Pascal’s triangle

Number of coin tosses (equal to row numbers in Pascal’s triangle)	Possible outcomes (H, heads; T, tails)	Number of possible outcomes in Pascal’s triangle
1	H	1
	T	1
2	HH	1
	HT, TH	2
	TT	1
3	HHH	1
	HHT, HTH, THH	3
	HTT, THT, TTH	3
	TTT	1
4	HHHH	1
	HHHT, HHTH, HTHH, THHH	4
	HHTT, HTHT, HTTH, THHT, THTH, TTHH	6
	HTTT, THTT, TTHT, TTTH	4
	TTTT	1

Impressively, even today, Blaise Pascal’s simple triangle can accurately predict probabilities for the Baseball World Series [18]. The World Series is played between two teams as “best out of 7” games. If the opposing team won the first game, there are six games remaining. Row 6 in the triangle depicts 64 (2^6^) possible outcomes (1-6-15-20-15-6-1). The home team will have to win at least four games to secure the title, whereas the opposing team requires only three more victories. There are 22 (1 + 6 + 15) possibilities for the home team to clinch the title (22/64 or 34.4%), compared to 42 (20-15-6-1) options for the opposing team to win three or more games (42/64 or 65.6%). Thus, by carrying a copy of Pascal’s antiquated triangle from 1653 in the pocket, a modern person of the twenty-first century can place a safe bet at the sports bar.

## Intuitive versus deliberate decision-making in critical conditions

A significant portion of the important aspects of clinical decision-making in critical care and surgical disciplines is acquired through iterative experiential learning. The famous Russian-American novelist and philosopher Ayn Rand stated in her 1964 essay “The Virtue of Selfishness” [19]: “Rationality is man’s basic virtue, the source of all his other virtues. Man’s basic vice, the source of all his evils, is the act of unfocusing his mind, the suspension of his consciousness, which is not blindness, but the refusal to see, not ignorance, but the refusal to know. Irrationality is the rejection of man’s means of survival and, therefore, a commitment to a course of blind destruction; that which is anti-mind, is anti-life.” Over a career, the master surgeon and critical care clinician typically develop reliable heuristics or “rules-of-thumb” that facilitate intuitive, efficient, reliable, and effective care. How often though, when adverse events occur in the operating room or intensive care unit, is the reflexive response to seek a causal explanation related to individual error, instead of questioning the care systems and organizational factors (both human and structural) that created the environment where preventable errors could have been mitigated or prevented? Magda Osman, the author of “Future-minded” (2014), stated the following applicable quote [20]: “Coincidences are the product of rational cognitive processes, and are an unavoidable result of our mind searching for causality in reality.” In his monumental opus, “Thinking Fast and Slow” [21], Daniel Kahneman identified the neurocognitive basis for *System 1* (intuitive) and *System 2* (deliberate) thinking that informed decision-making is relevant to safety and risk and is highly applicable to domains of surgery and acute care. *System 1* thinking is informative as to why complex problems are framed in isolation “where decisions are shaped by inconsequential features of choice problems” [21]. In contrast, *System 2* thinking—while working synergistically with *System 1*—is often less effectively accessed in the time-constrained and highly pressured clinical environments of an operating room or intensive care unit. Kahneman stated in his famous work [21]: “The division of labor between System 1 and System 2 is highly efficient: it minimizes effort and optimizes performance. The arrangement works well most of the time because System 1 is generally very good at what it does: its models of familiar situations are accurate, its short-term predictions are usually accurate as well, and its initial reactions to challenges are swift and generally appropriate. (…) One main limitation of System 1 is that it cannot be turned off.”

Intuitive *System 1* thinking has been referred to in more a more colloquial sense as “gut responses.” The appreciation and respect for the impulse-driven actions of a surgeon or intensivist in the midst of a medical crisis are essential and acquired over years of training and clinical experience. However, it is precisely this reliance on personal expertise, mastery, and heuristic dependence in place of deliberative, evidence-based *System 2* reflection potentially increases the risk for medical errors. Often, it is this mind-set of empirical practice that leads surgeons and acute care clinicians to embrace “personalized” anecdotal clinical decision-making over protocolized, evidence-based systematic care. Buchanan and O’Connell pointedly stated in their excellent article in the *Harvard Business Review* in 2006 [22]: “We don’t admire gut decision makers for the quality of their decisions so much as for their courage in making them. Gut decisions testify to the confidence of the decision maker, an invaluable trait in a leader. Gut decisions are made in moments of crisis when there is no time to weigh arguments and calculate the probability of every outcome. They are made in situations where there is no precedent and consequently little evidence. Sometimes they are made in defiance of the evidence.”

Loss aversion, a cornerstone concept in behavioral economics, highlights that even the most rational people value potential loss over potential gain in most scenarios. Kahneman has cogently argued that loss aversion is a hard-wired concept [21]. This neurocognitive response is driven by the basic adrenergic response of “fight, fright, or flight” and accounts and in large part for what Dan Ariely coined the term “sunk cost fallacy” [23]. This entails specifically that in any transaction, including medical decision-making, the pain of a projected loss factors strongly into predictably irrational behavior that can thwart optimal outcomes [23]. In the case of surgical or acute care decision-making, the potential for immediate substantial effort (such as a prolonged high-risk operation) or harm weighs more heavily than future potential benefit or opportunity for risk-reduction—even in the face of evidence that rationally argues against such a decision. The loss-aversive thresholds for positive and negative stimuli and their consequences evolve with maturity and professional experience, as illustrated by early childhood development [23]. The same is arguably true for surgeons and acute care physicians in-training. One may speculate that the inability to change behavior in complex healthcare systems, such as operating rooms, intensive care units, and emergency departments, may in part be a consequence of iterative loss-aversion behavior. Surgeons and acute care clinicians are best capable of anticipating and preparing for the potentially negative effects of *System 1* thinking, including the predictably irrational loss aversive behavior [23], by consciously rehearsing and formally simulating the full range of potential scenarios that could arise in their care environment. “Opt outs” and “nudges” are two potentially powerful behavioral economic interventions that can be designed to mitigate the negative impacts of loss-aversive and other predictably irrational behaviors to which surgeons and acute care clinicians are prone. The “buy-in” into surgical checklists and standard work care protocols are examples of interventions that have been shown to be highly effective in reducing operating room and intensive care risk and preventable patient harm [24].

## A deliberate decision-making strategy to minimize risk in surgery

The evolving understanding of probabilities since the seventeenth century that allowed predicting future events and the mastery of risk represent the main catalysts that developed our current modern society [25, 26]. Without risk-taking, our society would have remained deprived of a free economy with capital markets, banking, insurances, instant communication, speed travel, commercial aviation, and modern health care [2]. Beyond a doubt, the field of surgery remains a “risky business.” For surgeons, understanding risk assessment, risk stratification, and risk management appears imperative as a core responsibility of the surgical profession [26]. By understanding risk and probability, surgeons are empowered to more coherently counsel their patients. The former Johns Hopkins neurosurgeon and recent presidential candidate, Dr. Ben Carson, coherently stated that minimizing risk is frequently the only valid option for surgeons. Dr. Carson provided a pragmatic and clinically practicable solution to surgical decision-making in the face of risk and uncertainty. He suggested to use a checklist with four specific questions as a standardized deliberate decision-making approach to help determine whether it is worth taking a calculated risk [27]:

1. “What is the *best* thing that can happen if I take the risk?”
2. “What is the *worst* thing that can happen if I take the risk?”
3. “What is the *best* thing that can happen if I don’t take the risk?”
4. “What is the *worst* thing that can happen if I don’t take the risk?”

In analogy to other high-risk industries, such as aerospace engineering, our modern age of risk-taking is driven by our ability to take a calculated risk. Arthur Rudolph (1906–1996), who was a German rocket engineer during World War II who later developed the Pershing missile and Saturn V rocket for NASA, stated after the successful moon landing in 1969: “You want a valve that does not leak and you try everything possible to develop one. But the real world provides you with leaky valves. You just have to determine how much leaking you can tolerate.”

## Conclusions

Understanding the impressive and fairly young history of risk and probability, which evolved in just the past 300 years and redefined our modern society, will allow surgeons and patients to have a more honest and transparent discussion on the risks and benefits of surgical procedures as part of the preoperative informed consent. Clearly, the “quest for zero risk” remains a common fallacy in our society [27]. Thus, minimizing risk and defining an “acceptable risk” for patients frequently remain the few pragmatic options as part of the shared decision-making process for surgery [24, 28, 29].
